# Local immunization impacts the response of dairy cows to *Escherichia coli* mastitis

**DOI:** 10.1038/s41598-017-03724-7

**Published:** 2017-06-13

**Authors:** Vincent Herry, Christophe Gitton, Guillaume Tabouret, Maryline Répérant, Laurine Forge, Christian Tasca, Florence B. Gilbert, Edouard Guitton, Céline Barc, Christophe Staub, David G. E. Smith, Pierre Germon, Gilles Foucras, Pascal Rainard

**Affiliations:** 1 IHAP, Université de Toulouse, INRA, ENVT, Toulouse, France; 2 ISP, INRA, Université de Tours, UMR1282, Nouzilly, France; 3 PFIE, INRA, UE1277, Nouzilly, France; 4 UEPAO, INRA, UE1297, Nouzilly, France; 5 Institute of Biological Chemistry, Biophysics and Bioengineering, Heriot-Watt University, Edinburgh, UK EH14 4AS

## Abstract

Current vaccines to *Escherichia coli* mastitis have shown some albeit limited efficacy. Their mode of action has not been documented, and immune responses protecting the mammary gland against *E. coli* are not completely understood. To improve our knowledge of mammary gland immune protection, cows immunized either intramuscularly or intramammarily with the *E. coli* P4 were submitted to a homologous mastitis challenge. A third group of mock-immunized cows serve as challenge controls. Local immunization modified favorably the course of infection, by improving bacterial clearance while limiting inflammation. Systemic clinical signs and reduction in milk secretion were also contained. This occurred with a modification of the cytokine profile, such as an increase in IFN-γ and a reduction in TNF-α concentrations in milk. Concentrations of IL-17A and IL-22 increased in milk at the onset of the inflammatory response and remained high up to the elimination of bacteria, but concentrations did not differ between groups. Accelerated bacteriological cure was not linked to an increase in the initial efficiency of phagocytosis in milk. Results support the idea that antibodies did not play a major role in the improvement, and that cell-mediated immunity is the key to understanding *E. coli* vaccine-induced protection of the mammary gland.

## Introduction

Mastitis, a result of infection of the mammary gland, is the major disease of dairy cows, and *Escherichia coli* is one of the main causative pathogens of clinical mastitis. Due to the severity of most cases, *E. coli* mastitis is a major economic and welfare issue in dairy cow husbandry. Many attempts have been made to improve defenses of the cow against *E. coli* mastitis, among which vaccines have been developed to this end. Current vaccines are based on the use of killed rough *E. coli* such as the J5 strain. They show some efficiency in reducing the incidence of clinical mastitis and milk losses upon natural exposure of dairy cows, and in reducing the severity of mastitis in some but not all of the experimentally induced mastitis trials^1^. The mechanisms by which vaccination achieves these results are not convincingly identified. What is known is that neutrophils are critical to the control of infection and that a favorable outcome of *E. coli* mastitis depends on the prompt recruitment of active neutrophils into the mammary gland and milk^2, 3^. The role of antibodies is disputed^4^.

It has been speculated that vaccination would operate through the reinforcement of neutrophil recruitment by T helper type 1 (Th1) lymphocytes, although this has not been documented^4^. It appears that a significant challenge to successful vaccine improvement is our poor understanding of the immune responses that correlate with protection against coliform mastitis.

Since the development of commercial mastitis vaccines, research has unveiled the eminent role of the cytokine IL-17 in neutrophilic inflammation, and of the Th17 cells in orchestrating defenses of epithelial borders against infection by extracellular bacteria and fungi^5, 6^. Recently it has been shown with mouse mastitis models that IL-17A and Th17 cells are instrumental in the defense of the mammary gland against infection by *E. coli* or *Staphylococcus aureus* ^7, 8^. It is also possible to induce an antigen-specific neutrophilic inflammation in the bovine mammary gland which correlates with the production of IL-17A and IFN-γ and with circulating Th17 cells and the presence of IL-17A in milk^9^. Nevertheless, the role of IL-17 in bovine *E. coli* mastitis remains speculative, and the capacity to mobilize the Th17-immune axis by immunization and its impact on the course of infection have not been investigated.

To improve our knowledge of the protective immune response against mammary gland *E*. *coli* infections, we devised an experiment that involved immunization of dairy cows before their challenge with a mastitis-causing *E. coli* isolate. The immunization protocol was a prime-boost strategy with two different approaches; priming was done by the systemic (intramuscular) route in both cases, boosting was done either using the same intramuscular injection or by infusion of *E*. *coli* antigens in the teat canal.

The adjuvant was chosen on the basis of its capacity to induce a cell-mediated response in cows including circulating CD4 + lymphocytes producing IL-17A and/or IFN-γ^9^. The protective effects of the two different protocols were assessed in an homologous challenge. The results indicate that the course of infection was modified differently by the two immunization regimes. The cytokine profile of the inflammatory response was altered, with some unexpected results. Overall, the results confirm that cell-mediated responses induced by vaccination are more important than the humoral response to improve the response of the mammary gland to *E. coli* infection.

## Methods

### Ethics Statement

All procedures involving animals received approval from the Ethics Committee of Val de Loire (France), DGRI’s agreement APAFIS#813-2015061109103810v2. Animal studies were compliant with all applicable provisions established by the European directive 2010/63/UE. All methods were performed by approved staff members in accordance with the relevant standard operating procedures approved by the above mentioned ethics committee. All animals used in this study were handled in strict accordance with good clinical practices and all efforts were made to minimize suffering.

### Animals and study design

Eighteen Holstein-Frisian heifers were purchased in two commercial farms at about 1 year old, and were then raised together in the same barn with deep straw bedding at the INRA animal facility (PFIE, Nouzilly), and fed a diet covering the dietary requirements at each physiological stage (French feeding table^10^). They were synchronized and inseminated at 15 month-old on average. Two months before the anticipated date of calving, heifers were allocated in two groups according to the expected date of calving, and housed, fed and managed identically. One month before the anticipated calving date, ration was progressively (in a one week period) changed from a hay-based ration with concentrate to a diet based on corn silage, hay, soybean meal, and a concentrate (soybean, wheat, barley and corn grain) formulated to meet the dietary requirements for the transition dairy cow and early lactation^10^. During the precalving period, trace mineral status of the heifers was assessed for selenium, zinc and copper by dosing glutathione peroxidase activity in erythrocyte and zinc and copper plasma concentrations, respectively. All results were within the reference range. After calving, the lack of postpartum diseases (i.e. retained foetal membranes and endometritis) was monitored, and subclinical ketosis (defined as β-hydroxybutyrate (BHBA) > 1.4 mM) was assessed by regular detection of blood BHBA (Optium Xceed, Abott). In the postcalving period and throughout the experiment, the cows were milked twice a day (at 08h00 and 16h00) by experienced and highly-qualified animal keepers in a milking parlor adjacent to the barn. Forestripping, pre and postdipping were implemented. Beginning one week before challenge to the whole study period, the four-quarter production was weighed in the same bucket.

### Immunogenic preparations

The *E. coli* strain P4 was used for the two immunogenic preparations and intramammary challenge. This strain has been isolated from a case of acute bovine mastitis and used extensively in experimentally induced mammary gland infections^11–14^. Strain P4 is classified as O32:H37, ECOR phylogenetic group A, and multilocus sequence type ST10^15^.

Heat-killed bacteria (HKEc) were prepared by culture in RPMI1640 overnight at 37 °C under moderate agitation (150 rpm). Bacteria were harvested by centrifugation (3,500 x *g* at 20 °C for 15 min), and resuspended in PBS. Concentration of bacteria was estimated from the OD (540 nm) of the overnight culture and retrospective cfu number determination (by plating on LB agar plates). Bacteria were killed by heating at 60 °C for 60 min in vials that were completely immersed in a water bath. Absence of viable bacteria was checked by plating on LB agar and overnight incubation at 37 °C. For intramuscular injections, HKEc (2 × 10^9^ killed bacteria per dose) were emulsified in oil adjuvant (Montanide ISA 61VG®, Seppic, France) in the proportion indicated by the manufacturer.

To prepare *E. coli* culture supernatant (SNEc), *E. coli* strain P4 was grown in the same conditions, and bacteria were spun down by centrifugation at 10,000 x *g* for 20 min at 4 °C. The culture supernatant was filtered (0.45 µm) to remove bacterial cells, diafiltered and concentrated with a tangential flow membrane system (Vivaflow, 10,000 molecular weight cutoff, Sartorius). The concentrate was finally sterile-filtered on 0.45 µm pore membrane to minimize the retention of outer membrane vesicles, and stored in portions at −80 °C. Protein content equivalent was determined with micro BCA protein assay kit (ThermoFisher Scientific). Immunization doses contained 50 µg of protein per quarter.

The use of SNEc in the intramammary immunization was to favor the immune response against *E. coli* proteins, and more specifically proteins of the outer membrane and periplasm. For the intramuscular booster injection, we decided to keep killed whole bacteria as a base of comparison for the orientation of the response to surface proteins, which shall be analyzed in a further study.

### Immunization Scheme

**Figure 1:**
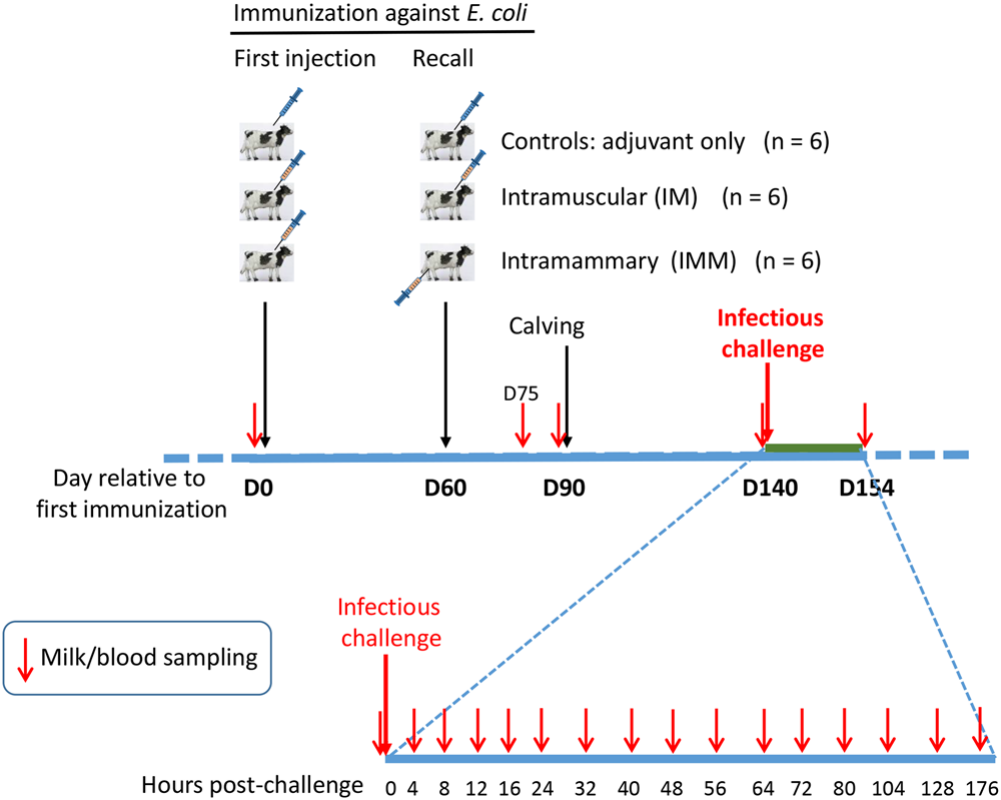
Experimental scheme. Three groups of six cows were immunized before calving by using three different immunization schedules. About two months after calving, all cows were challenged by inoculation of *E. coli* strain P4 into one quarter. Milk and blood samplings were carried out at the indicated times post-inoculation to monitor the infection and the immune response.

The experiment comprised two identical waves of nine cows. In total, three groups of six cows that were randomly assigned to one of the following immunization schedules (Fig. 1):

1. Intramuscular injections (**IM**; n = 6). Cows received two IM injections of HKEc P4 in Montanide adjuvant two months apart, at three months and one month before calving.
2. Intramammary boost (**IMM**; n = 6). Cows received intramuscular injection of the immunisation preparation (HKEc) three months before calving and an intramammary injection of SNEc one month before calving.
3. **Control** group (n = 6) was injected with adjuvant by the intramuscular route twice at the same dates.

Three cows from each treatment group were in each challenge wave. Intramuscular injections (2 mL) were done in the upper part of the caudal neck just anterior to the scapula using 21 G needles. Intramammary infusions consisted of 1 mL of SNEc injected via the teat canal into the four glands of the udder using a sterile smooth cannula fitted to a 1-mL syringe. Before injection, teat apices were thoroughly scrubbed with cotton-wool soaked in 70% alcohol. After injection, a teat sealer (OrbeSeal®, Zoetis) was infused into each quarter.

Following immunization, cows were daily monitored for signs of general and local reactions to the immunization by animal keepers, and temperature was recorded using an intraruminal device (see below).

### Intramammary Bacterial Challenge

Cows were challenged at 44 to 56 (average 49) days in milk. Before challenge, all quarters were checked for the absence of intramammary infections by performing bacteriological analysis and SCC measurement on milk. Preferably, a hind quarter was challenged, with the other hind quarter and the front quarters serving as within-cow controls. Inoculated quarters were free of infection: their milk had less than 50,000 cells per mL and was exempt of cultivable bacteria.

To prepare the inoculum, *E. coli* P4 was grown overnight in Brain Heart Infusion broth at 37 °C at rest. Bacteria were collected by centrifugation and washed once in pyrogen-free Dulbecco’s PBS (DPBS; Lonza, Verviers, Belgium). Bacteria were then diluted in DPBS and adjusted to the concentration of 1000 cfu/mL by measuring the optical density at 600 nm. One quarter of each cow was challenged by infusion of 1 mL of the bacterial suspension. Portions of the inoculum suspension used to challenge the cows were returned to the laboratory to verify the purity of the challenge solution and viability of the microorganisms. This suspension was plated on blood agar, incubated at 37 °C, and counted to verify the actual cfu/ml that was used for inoculation. The results were 1040 cfu for the first wave and 750 cfu for the second wave of inoculations. Inoculation was carried out just after complete milking of the gland, and 8 h before the next milking. Complete milkings subsequent to inoculation took place twice a day, at 8, 16, 32, 40, 56, 64, 80, 88, 104 hpi and so on.

### Assessment of systemic and local clinical response to infection

Systemic and local mammary signs were assessed through the trial period as described by Wenz *et al*. (2006) by two experienced veterinarians unaware of the cows group allocation. Clinical signs were recorded the day before inoculation and at 4, 8, 12, 16, 24, 32, 40, 56, 64 and 80 hours post-inoculation (hpi). Systemic signs were scored on a 9-point scale: 0–2 mild or no disease, 3–5 moderate disease, 6–9 severe disease. Rectal temperature, hydration status, rumen contraction rate and general attitude (signs of depression) were recorded. A bolus (Thermobolus, Medria Elevage, France) recording intraruminal temperature every 30 minutes was inserted into the rumen of each cow at the beginning of the experimental design.

Mammary signs were scored on a 7-point scale: 0–2 mild or no disease, 3–4 moderate disease and 5–7 severe disease. The mammary gland was observed and palpated for swelling, firmness, pain and milk appearance. Milk production was weighed twice a day for each cow, but individual quarter production was not assessed.

Ultrasonography of the mammary gland was performed by the same operators throughout the experiments using an Esaote Piemedical MyLab30 ultrasound device equipped with a linear LA332 probe (Hospimedi France, Valdampierre, France). Ultrasonography of udder glands was performed at 5 MHz immediately after milking by placing the probes along the udder skin, taking great care not to flatten the lactiferous ducts and cisterns. Data were acquired on the four quarters before challenge and only uninflamed quarters were considered suitable for the experiment. Ultrasonography measurements were carried out on the challenged and contralateral quarters just before challenge and at 8, 16, 32, 40, 56, 80 and 208 hours post infusion. Results were given as ratio (in %) of the inflamed area to the scanned mammary gland surface.

### Sample collection and records

Milk samples were collected aseptically (after scrubbing the teat apex with cotton wool moistened with 70% alcohol) in sterile vials for microbiological culture, total and differential somatic cell count (SCC) on the day before and at 4, 8, 12, 16, 24, 32, 40, 48, 56, 64, 80, 88, 104, 128 and 176 hpi. Only the inoculated quarters were sampled and the samples were immediately transported on ice to the laboratory. Blood samples from the jugular vein were collected in EDTA evacuated tubes (Venosafe™, Terumo® Europe) from all cows at the following time points: prior to inoculation and at 4, 8, 12, 16, 24, 32, 40, 56, 64, 80 hpi.

### Milk leukocytes and bacterial counts

SCC were determined from aseptically taken foremilk samples from the challenged quarters using a cell counter (Fossomatic model 90; Foss Food Technology, Hillerod, Denmak) as described^16^. Foremilk samples from infected glands which had high cell numbers were diluted 1:10 in PBS before analysis. Differential milk SCC were acquired as previously described^17^. Briefly, samples were collected v-v in Alsever’s solution and centrifuged for 15 min at 800 × *g* and 4 °C. The cell pellet was washed twice in PBS, 20 mM EDTA and 20% Alsever’s solution before incubation with a combination of 5 µM SYTO 13 and 5 µM Vybrant DyeCycle Ruby stain (Invitrogen) in HBSS for 30 min in the dark at room temperature. Flow cytometry acquisitions were performed with a FACScalibur (Becton Dickinson). Data were analyzed using FlowJo software v10 (TreeStar, CA, USA). DNA positive events were gated based on the Vybrant DyeCycle Ruby stain intensity and cell types determined based on side scatter and SYTO-13 labeling. Milk total polymorphonuclear neutrophils (PMN), dead PMN and lymphocytes were then calculated back by multiplying the percentages of each population by the SCC. Finally, SCC were expressed as somatic cell score SCS = 3 + log2(SCC/100,000), (Ali and Shook, 1980).

Bacteriological analysis was performed throughout the experiment by plating 50 µL of tenfold dilutions of foremilk samples over sheep blood-esculin agar, overnight incubation at 37 °C, and *E. coli* colonies enumeration.

### Assessment of hematological and cellular parameters

Sixty microliters of whole blood were incubated with two different cocktails of pre-conjugated monoclonal antibodies for 20 minutes at room temperature in the dark. The first cocktail was used to stain bovine T lymphocytes and consisted in a combination of CD45-Pacific Blue (Biorad, USA, clone CC1), CD3-A680 (Kingfisher, USA, clone MM1a), CD4-RPE (Biorad, USA, clone CC8) and WC1-FITC (Biorad, USA, clone CC101) for TCRγδ T cells. The frequency of CD8 T cells was estimated by gating CD4^neg^ cells within the CD45^pos^ CD3^pos^ gate. A second cocktail was used to stain B cells, monocytes and granulocytes with CD21-PE (Biorad, USA, clone CC21), CD14-FITC (Biorad, USA, clone CC-G33) and CD172a-RPE Cy5.5 (Biorad, USA, clone CC149), respectively. Red blood cells lysis was achieved by adding 500 µL of MACS RBC Lysis Solution (Miltenyi Biotec) for 10 minutes. The osmolarity of the solution was restored using FACS buffer (Dulbecco’s modified PBS calcium and magnesium free, 2.5 mM EDTA, 0.1% BSA, pH 7.2) to a final volume of 1.2 mL thus giving a 1:20 dilution of the original blood volume. Samples were centrifuged at 300 × *g* for 5 minutes. The pellet was then suspended in 50 µL paraformaldehyde 4% in PBS, and incubated for 10 minutes at room temperature. Finally, FACS buffer was added to bring the total volume back to 1.2 mL. Data acquisition was made on 10,000 events using a MACSQuant Analyzer (Miltenyi Biotec) capable of absolute cell count, and data analysis was made with FlowJo software v10.

### Cytokines and inflammatory proteins ELISAs

Enzyme-linked immunosorbent assays (ELISAs) for the complement fragment C5a, the chemokines CXCL3 and CXCL8, TNF-α and IL-17A were performed as described^18–21^. Commercially available kits were used for bovine IFN-γ (Mabtech AB, Nacka Strand, Sweden), bovine IL-1β and IL-6 (Thermo Scientific, Rockford, IL, USA), bovine IL-10 (Bio-Rad AbD Serotec), bovine lactoferrin and serumalbumin (Bethyl Laboratories), and bovine serum amyloid A (SAA ELISA kit, Tridelta Development Ltd, Ireland). The concentration of plasma haptoglobin was determined by immunoprecipitation^22^. The ELISA for bovine IL-22 was developed in-house. Recombinant bovine IL-22 (bovine IL-22 ORF minus the signal sequence, with a hexahistidine tag) was expressed with the *Drosophila* Schneider S2 expression system by transfection with the pMT-PURO plasmid as described^23^. The protein was purified by metal affinity chromatography (Ni-NTA superflow column, Qiagen) as described^24^ and used to elicit antibodies in rabbits (Eurogentec, Liège, Belgium). Antibodies to IL-22 were affinity-purified by passage over a column of IL-22 coupled to EAH-Sepharose (GE Healthcare Life Sciences), before removal of antibodies to the tag by passage over a column of the tag sequence peptide coupled to EAH-Sepharose gel. Part of the antibodies were biotinylated with sNHS-lc-biotin (Interchim, France). A sandwich ELISA was developed by using the purified anti-IL-22 antibodies as capture antibodies (1.5 µg/mL) and biotinylated anti-IL-22 antibodies (1 µg/mL) as detection antibodies. Concentrations of IL-22 in milk samples were determined by reference to the standard curve with purified IL-22 (3200 to 100 pg/mL). The lower limit of detection was about 50 pg/mL.

### Assessment of antibody and cellular responses to immunization

Specific antibody titers were assessed with an ELISA using P4 *E. coli* whole cell as antigen. Flat-bottomed, 96-well microtitration plates (Certified Maxisorp Nunc Immuno Plate, Thermo Scientific) were coated with 1 µg/mL poly-L-lysine (Sigma) in PBS for 2 h at room temperature. Plates were then coated overnight at 4 °C with a heat-killed suspension (100 µL/well) of *E. coli* P4 (OD 0.2 at 620 nm) in PBS half-diluted with deionized water. After careful removal of 50 µL of PBS with a multichannel pipette, the plates were let to dry in an incubator at 37 °C and stored at 4 °C. These precautions ensured a firm adhesion of bacteria to the bottom of the wells. Before use, the plates were blocked with 0.5% (w/v) fish skin gelatin (Sigma) in PBS (PBS-G) for 30 min at 37 °C and washed with PBS containing 0.05% Tween 20 (PBS-T). The sequence of incubation steps (1 h at 37 °C) of analytes and reagents (100 µL/well), separated by three washes with PBS-T (MW 96/384 Microplate Washer, Beckman Coulter) was as follows for the detection of antibodies in the IgM and IgG_2_ isotypes: 1) an appropriate dilution of serum in PBS-G (1/100 to 1/2000); 2) 1/10,000 dilution of peroxidase conjugated sheep anti-bovine IgG_2_ or anti-bovine IgM (Bio-Rad AbD Serotec); 3) 100 µL of the chromogenic reagent tetramethylbenzidine (TMB; Uptima, Interchim) and finally 50 µL of stop solution (1 M HCl). The OD at 450 nm was read with a Multiscan RC reader (Labsystems). Antibody titers were given in arbitrary units. In each plate a series of dilution of a serum from an IM immunized cow was incorporated in duplicate. This serum was attributed a titer of 1000 arbitrary units of IgM or IgG_2_. Titers of sera under tests were calculated by the software (Genesis, Labsystems) by reference to the obtained standard curve. This allowed us to quantify the relative variations of antibody reactivity following immunization.

The cell-mediated response to immunization was assessed with a whole blood antigenic assay (WBA), performed as previously described^9^ with a few modifications. Blood was collected from the jugular vein into evacuated 10-mL tubes coated with lithium-heparin as anticoagulant (Venosafe^TM^, Terumo® Europe). Samples were used within 4 h after collection. Stimulations were performed in triplicate by mixing in 96-well microplates (Falcon Microtest™, Becton Dickinson) 100 µL of blood with either 100 µL of culture medium (RPMI-1640 supplemented with 10% fetal bovine serum, 2 mM L-glutamine, 10 mM HEPES, penicillin-streptomycin, fungizone and 0.05 mM 2-mercaptoethanol) as negative control, 100 µL of pokeweed mitogen (2 µg/mL) as positive control, or 100 µL of HKEc suspension (4 × 10^6^ bacteria/well). The culture was incubated at 37 °C in a humidified atmosphere with 5% CO2 for 2 days. Supernatants were then harvested and stored at −20 °C in 96-well plastic storage plates (Greiner™ bio-one) until assayed for cytokine (IL-17A and IFN-γ) content.

### Opsonophagocytosis assay

The opsonic activity of milk and serum was assessed by incubating blood neutrophils with live *E. coli* P4 bacteria. Bacteria were grown overnight in BHI broth at 37 °C without agitation. After one wash in RPMI 1640 + 40 mM HEPES + 0.5 mg/mL BSA (pyrogen-free, Sigma) (RPMI-AH), bacteria were resuspended in RPMI-AH at the concentration of 2.5 × 10^6^ cfu/mL. Polymorphonuclear granulocytes (PMN) were isolated from blood samples taken from healthy cows as described^25^. Incubation of neutrophils with bacteria was performed in 96-well microplates (Tissue culture grade, Falcon) for 90 min at 37 °C during which granulocytes and bacteria were maintained in suspension by slow rotation of the plate on edge on a vertical turntable^26^. Mixtures of 20 µL of bacterial suspension and 40 µL of PMN suspension were distributed in the wells along with 20 µL of milk or serum dilution and 20 µL of RPMI-AH. Dilutions were determined by pilot experiments with a view to enabling the assay to distinguish opsonin sources of different efficiencies. Low final concentrations of 0.25% and 1% were used for serum, and 20% for milk. Control wells without opsonin source (negative control) or with 5% blood serum (pooled sera from control cows, positive control) were incorporated in each plate. At the end of the incubation period, 10 µL portions of the phagocytic mixture were transferred to wells containing 90 µL of PBS + 0.2% Triton X100. After vigorous homogenization, serial 1/10 dilutions were performed. Finally, 10 µL portions of three consecutive tenfold dilutions were deposited on BHI agar. Colony-forming units were counted after overnight culture at 30 °C.

### Assay of phagocytic bactericidal activity of whole fresh milk

Milk from infected quarters was taken at 12, 16 and 32 hpi with aseptic precautions. Within 2 hours after collection, 1 mL of milk was incubated under end-over-end agitation with 100 µL of *E. coli* P4 suspension (10^7^ cfu/mL) for 1 h at 37 °C. In parallel, a control tube containing the same phagocytic mixture was incubated at rest. An aliquot from each phagocytic mixture was serially diluted in PBS + 0.2% Triton X100 before plating on trypticase soy agar. After an overnight incubation at 37 °C, colony-forming units of each culture plate were enumerated. Bacterial survival percentage was calculated from the reduction in bacterial numbers in agitated tubes compared to bacterial numbers in tubes kept at rest.

### Statistical analysis

All data were expressed as the mean value ± SEM of n = 6 cows per group. Statistical analyses were performed using the GraphPad Prism 6.0 sofware by applying Two-way ANOVA with Bonferroni correction. Results were also tested using a Mann-Whitney test. The statistical significance was considered at p-value below 0.05.

### Data availability

Most of the data generated or analyzed during this study are included in this published article (and its Supplementary Information file). Other data are available from the corresponding author on reasonable request.

## Results

As the experimental infections have been performed in two waves, an analysis was performed to uncover a possible wave effect on the response to infection. A principal component analysis (PCA) involving eleven infection parameters was carried out with the web tool ClustVis^27^. The scatter plot in the Supplementary Fig. S1 shows that the cows from the two waves are distributed in superimposed clusters, indicating that there was no wave effect.

### Systemic signs and temperature

**Figure 2:**
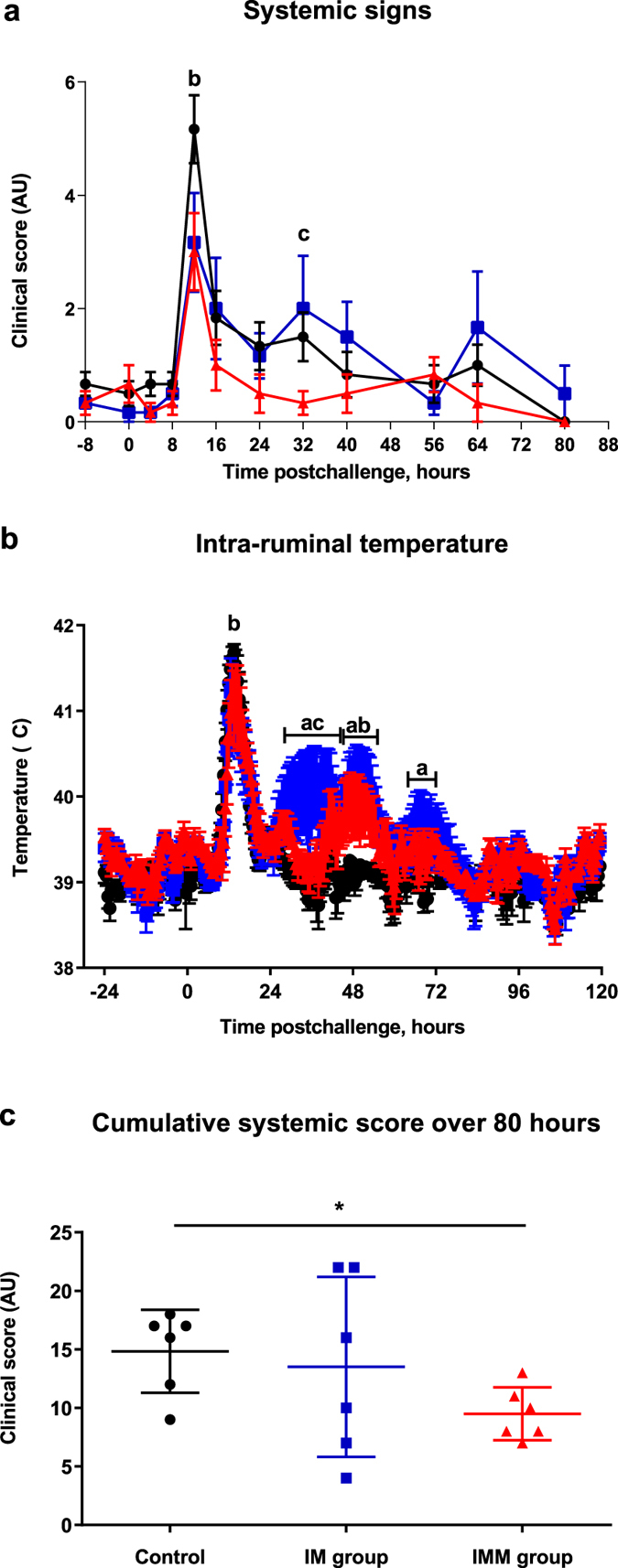
Systemic symptoms. Systemic clinical score (**a**), intra-ruminal temperature (**b**) following intramammary challenge with *E. col*i P4 (0 h) and cumulative systemic score (**c**) over the first 80 hours following intramammary challenge in first lactation heifers injected with adjuvant only (black circles), in heifers immunized with intramuscular injections only (blue squares) or immunized with intramuscular and intramammary injections (red triangles). Means and error bars, corresponding to standard error of the mean (a and b) or standard deviation (**c**), are presented. Letters (**a**,**b**,**c**) indicate a difference (p value < 0.05) between control and IM groups, control and IMM groups, and IM and IMM groups respectively. Asterisk indicate a difference (P < 0.05) between immunization groups.

Following inoculation, all cows developed systemic signs of mastitis classified as moderate to severe. At 12 hpi, systemic signs were significantly less severe in the IMM group than in the controls (p < 0.05, two-way ANOVA and Mann Whitney test) and tended to be milder for the IM group compared to the controls (Mann-Whitney test, p = 0.12) (Fig. 2a). Later, at 32 hpi, the IM group experienced a second phase of increased systemic signs, with systemic scores significantly higher than the IMM group (2 vs 0.33, p < 0.05, two-way ANOVA). This second clinical phase was also absent in the control group. A second and a third wave of hyperthermia (>40 °C) were observed for the IM group with a significantly higher temperature than the controls at 30–50 hpi and 68–76 hpi (Fig. 2b). The temperature was also higher in this group than in the IMM group at 30–40 hpi. The intra-ruminal temperature of the IMM group was significantly higher for a brief episode at 46–52 hpi compared to the controls (Two-way ANOVA, p < 0.05). At 56 hpi, all cows had returned to the original state with no or mild systemic signs. Considering the cumulative systemic score between groups over the 80-hour examination period, the overall severity of systemic signs was significantly lower for the IMM group compared to the control group (p = 0.02; Mann Whitney test) (Fig. 2c).

### Mammary signs and milk production

**Figure 3:**
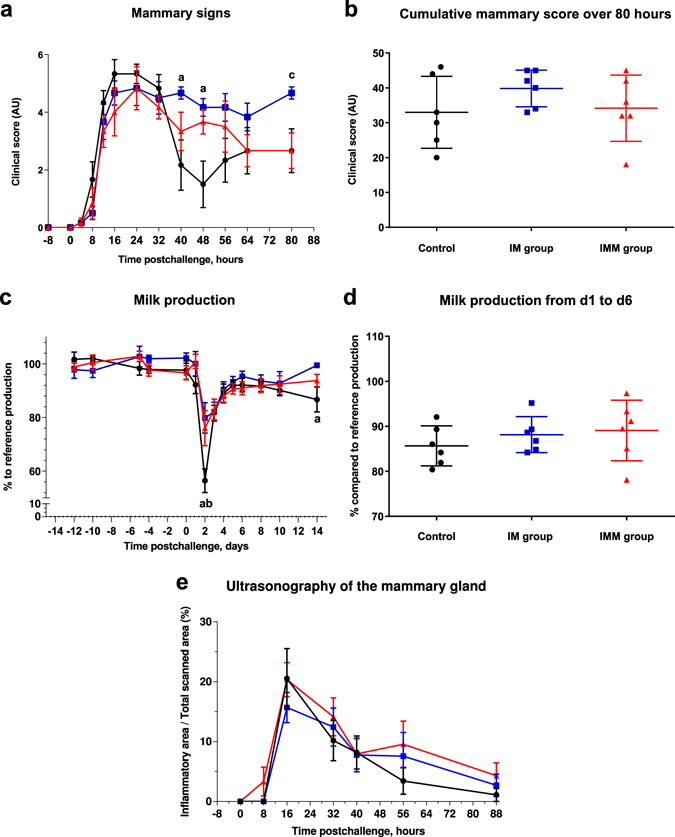
Local symptoms and milk production. Mammary clinical score (**a**), Cumulative local mammary score over the first 80 hours upon intramammary challenge (**b**), Daily production expressed as a percentage of the reference production before challenge (**c**), milk production from day 1 to 6 (% compared to reference production) (**d**) and subcutaneous inflammation of the udder gland (**e**) following intramammary challenge with *Escherichia coli* P4 at 0 h in first lactation heifers from three treatment groups: adjuvant only (black circles), intramuscular injections (blue squares) intramuscular (**c**) and intramammary injections (red triangles). Means and error bars, corresponding to standard errors of the mean for each treatment group at each sample point (**a** and **b**) or standard deviation (**c**,**d** and **e**), are presented. Letters (a,b,c) indicate a difference (p value < 0.05, Two-way ANOVA and/or Mann Whitney test) between control and IM groups, control and IMM groups, and IM and IMM groups respectively. Asterisk indicate a difference (P < 0.05, Two-way ANOVA and/or Mann Whitney test) between immunization groups.

Mammary signs of mastitis were identified in all cows and were detectable from 12 hours after challenge. During the acute phase of mastitis, local signs were not significantly different between groups (Fig. 3a). However, mammary clinical scores decreased from 40 to 80 hpi for the control and IMM group but remained high for the IM group that experienced a persistent local inflammation from 40 hpi to the end of the examination period (at 80 hours), with significantly higher mammary scores (p < 0.05; two-way ANOVA and Mann Whitney test) at 40 and 48 hpi compared to the controls, and at 80 hpi compared to the IMM group, respectively. When we compared the cumulative mammary score between groups over the 80-hour examination period, the overall severity of mammary signs was not significantly different between groups (Fig. 3b).

Milk production was reduced during the acute phase of infection (Fig. 3c). However, at day 2 pi, milk loss was significantly lower in the IM and IMM groups than in the controls (80%, 76% and 56.5% of the reference production before inoculation for the IM, IMM and control group, respectively (p < 0.05, two-way ANOVA and Mann-Whitney test). At day 14, the IM group had a significantly lower milk loss than the controls (p < 0.05; two-way ANOVA and Mann-Whitney test). Despite these differences, the overall change in milk production was not different between groups (Fig. 3d).

The ultrasonographic examination of mammary tissue revealed an extensive subcutaneous oedema of the udder of the 18 challenged cows, which was sizeable as soon as 16 hpi and persisted up to 56 hpi (Fig. 3e and Supplementary Fig. S2). Oedema disappeared completely within a week with udder gland parenchyma back to normal at 184 hpi (not shown). Overall, there was no difference between groups.

### Bacterial counts and duration of IMI

**Figure 4:**
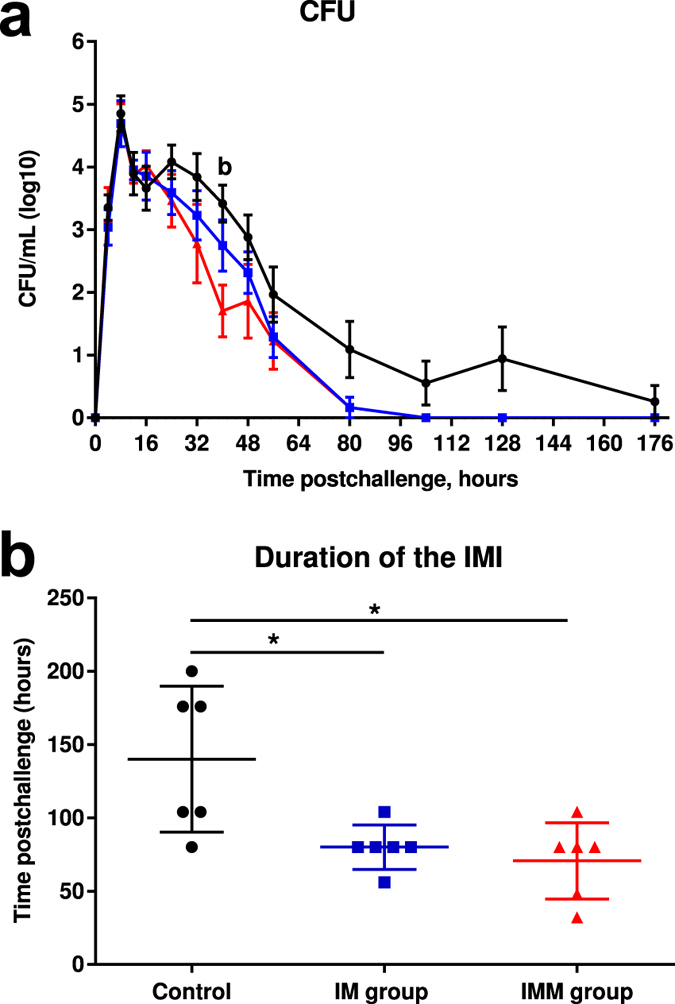
Infection monitoring. Colony forming units per mL of milk expressed as log_10_ (**a**) upon intramammary challenge with *E. coli* P4 at 0 h and duration of the intramammary infection (**b**) upon intramammary challenge in first-lactation heifers from the three treatment groups: adjuvant only (black circles) intramuscular injections (blue squares) intramuscular and intramammary injections (red triangles). Means and error bars, corresponding to standard errors of the mean for each treatment group at each sample point (**a**) or to standard deviation (**b**), are presented. Letters (a,b,c) within hours indicate a difference (P < 0.05, Two-way ANOVA and/or Mann Whitney test) between control and IM groups, control and IMM groups, and IM and IMM groups respectively. Asterisk indicate a difference (P < 0.05, Two-way ANOVA and/or Mann Whitney test) between immunization groups.

Mean bacterial counts for each experimental group peaked at 8 hpi (Fig. 4a). Milk bacterial counts from challenged quarters was lower in the IMM group compared to the controls at 40 hpi (140 cfu vs 1380 cfu; Mann Whitney Test, p < 0.05). Furthermore, the mean duration of IMI was significantly lower for the immunized groups (80 ± 6.2 hours and 71 ± 10.6 hours for the IM and IMM groups, respectively) than the controls (140 ± 20.3 hours) (p < 0.05, Man Whitney test), but it was not different between the immunized groups (Fig. 4b).

### Milk and blood leucocytes

**Figure 5:**
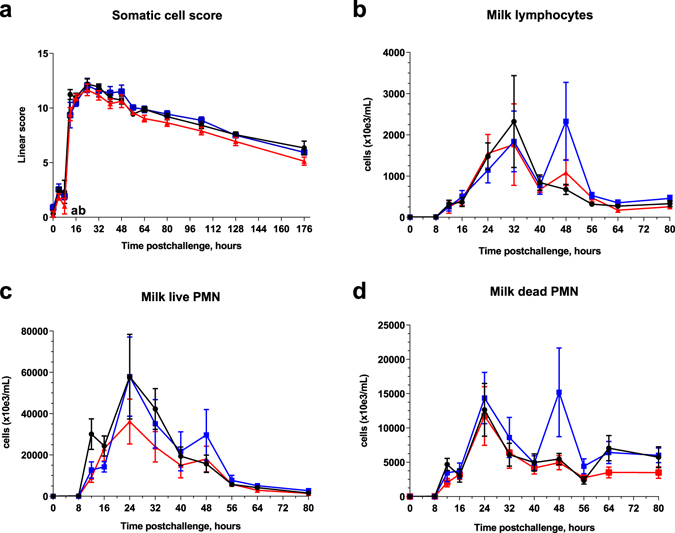
Milk leucocytosis monitoring. Linear score of somatic cell count (**a**), milk lymphocyte count (**b**), live PMN count (**c**) and dead PMN count (**d**) upon intramammary challenge with *E. coli* P4 at 0 h in first-lactation heifers from the three treatment groups: adjuvant only (black circles), intramuscular injections (blue squares), intramuscular and intramammary injections (red triangles). Means and error bars corresponding to standard errors of the mean for each treatment group at each sample point are presented. Letters (**a**,**b**,**c**) within hours indicate a difference (P < 0.05, two-way ANOVA and/or Mann Whitney test) between control and IM groups, control and IMM groups, and IM and IMM groups respectively. Asterisk indicate a difference (P < 0.05, Two-way ANOVA and/or Mann Whitney test) between immunization groups.

**Figure 6:**
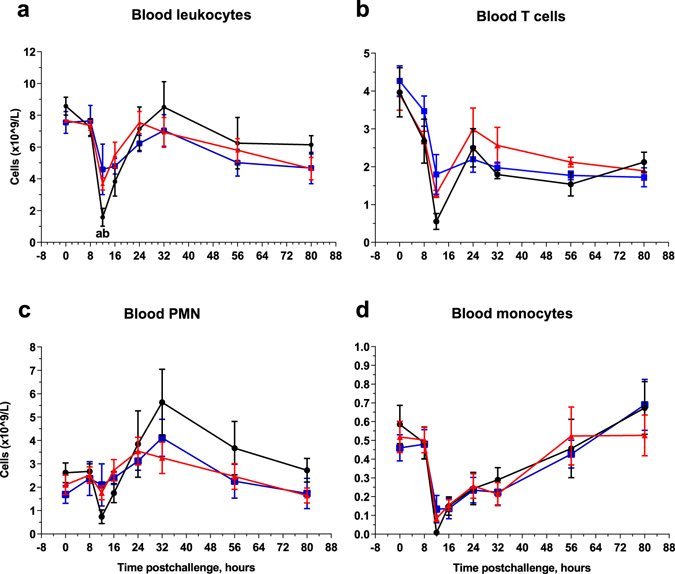
Blood leukocyte count. Total blood leucocytes (**a**), T lymphocyte count (**b**), PMN count (**c**), and blood monocyte count (**d**) upon intramammary challenge with *E. coli* P4 at 0 h in first-lactation heifers from the three treatment groups: adjuvant only (black circles), intramuscular injections (blue squares) intramuscular and intramammary (red triangles). Means and error bars corresponding to standard errors of the mean for each treatment group at each sample point are presented. Letters (**a**,**b**,**c**) within hours indicate a difference (P < 0.05, Two-way ANOVA and/or Mann Whitney test) between control and IM groups, control and IMM groups, and IM and IMM groups respectively. Asterisk indicate a difference (P < 0.05, Two-way ANOVA and/or Mann Whitney test) between immunization groups.

SCC increased dramatically at 12 hpi and peaked at 24 hpi for the three groups (Fig. 5a). However, at 12 hpi, linear score of SCC was lower for the immunized cows (Two-way ANOVA, p < 0.05). At 48 hpi, a second peak in milk lymphocytes and neutrophils (dead and alive neutrophils) was observed for the IM group compared to the IMM and control groups (non-significant; Fig. 5b and c). Percentages of dead neutrophils were similar in all groups except for the IM group at 48 hpi (Fig. 5d). At 12 hpi, all cows experienced a marked drop in blood leukocyte counts (Fig. 6a). However, leukopenia was less pronounced in the immunized IM and IMM groups than in the control group (Mann Whitney test, p < 0.05). Leukopenia was associated with a decrease in lymphocytes, neutrophils (PMN) and monocytes (Fig. 6b,c and d).

### Markers of blood and milk inflammation

**Figure 7:**
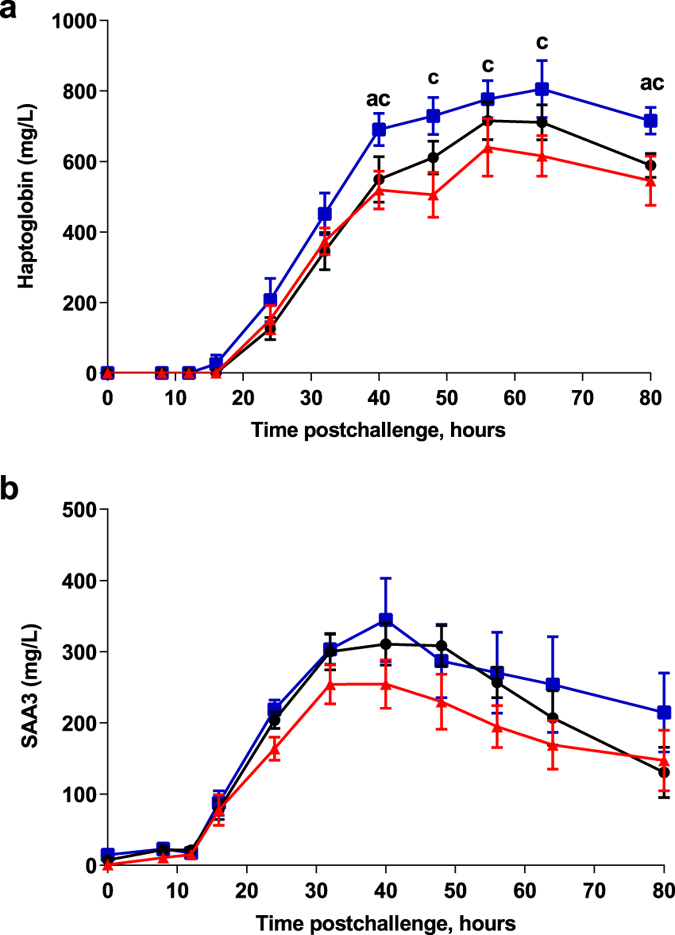
Systemic acute phase response. Plasma haptoglobin (**a**) and SAA3 concentration (**b**) upon intramammary challenge with *E. coli* P4 at 0 h in first-lactation heifers from the three treatment groups: adjuvant only (black circles), intramuscular injections (blue squares) intramuscular and intramammary injections (red triangles). Means and error bars corresponding to standard errors of the mean for each treatment group at each sample point are presented. Letters (**a**,**b**,**c**) within hours indicate a difference (P < 0.05, Two-way ANOVA and/or Mann Whitney test) between control and IM groups, control and IMM groups, and IM and IMM groups respectively. Asterisk indicate a difference (P < 0.05, Two-way ANOVA and/or Mann Whitney test) between immunization groups.

Two acute phase proteins were measured in the plasma of challenged cows. Haptoglobin was significantly higher in the IM group compared to the controls at 40 and 80 hpi (p < 0.05, two-way ANOVA) and higher in the IM group compared to the IMM group at 40, 48 hpi (two-way ANOVA and Mann Whitney test, p < 0.05) and at 56, 64 and 80 hpi (p < 0.05, p < 0.001 and p < 0.01 respectively, Two-way ANOVA) (Fig. 7a). Serum amyloid-A (SAA) concentrations tended to be the least in the IMM group, but the difference was not significant (Fig. 7b).

**Figure 8:**
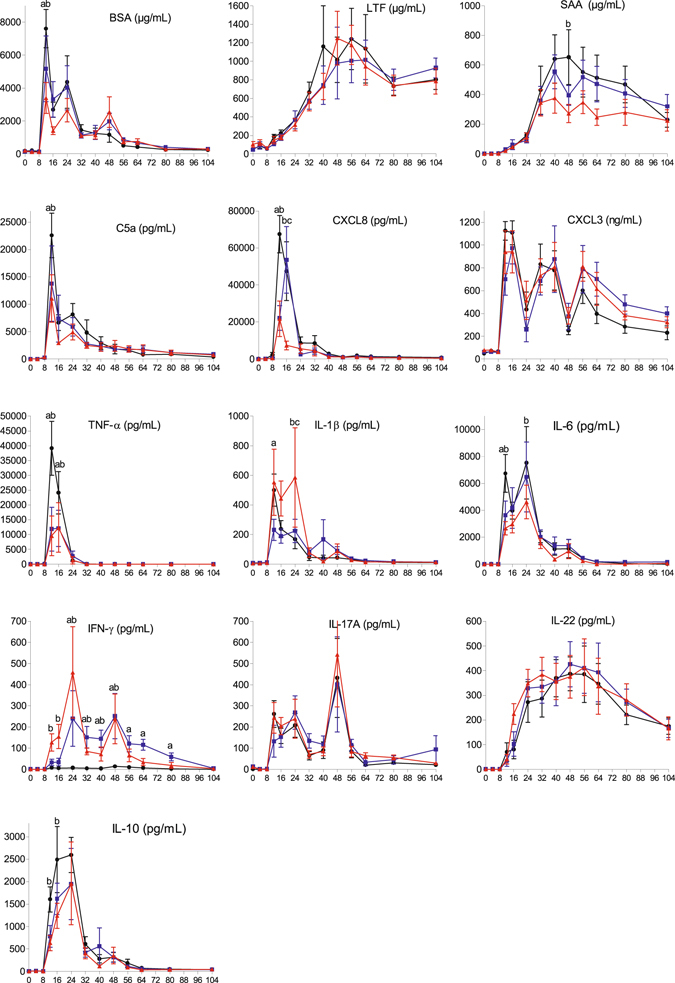
Indicators of inflammation and immune response in milk. Milk concentration of BSA, C5a, CXCL3, CXCL8, lactoferrin (LTF), serum amyloid A (SAA) and different cytokines in milk upon intramammary challenge in first-lactation heifers from the three treatment groups: adjuvant only (black circles), intramuscular injections (blue squares), intramuscular and intramammary injections (red triangles). Means and error bars corresponding to standard errors of the mean for each treatment group at each sample point are presented. Letters (**a**,**b**,**c**) within hours indicate a difference (P < 0.05, Two-way ANOVA and/or Mann Whitney test) between control and IM groups, control and IMM groups, and IM and IMM groups respectively. Asterisk indicate a difference (P < 0.05, Two-way ANOVA and/or Mann Whitney test) between immunization groups.

In milk, none of the measured indicators increased during the first 8 hpi, but most of them showed an abrupt increase by 12 hpi, indicating that the local inflammatory response was triggered shortly before that time (Fig. 8). We measured BSA concentrations as a marker of blood plasma exudation (breach of the milk-blood barrier). There were two peaks at 12 and 24 hpi for all cows, with a third peak at 48 hpi for the immunized cows only. The peak at 12 hpi was significantly lower in the IM and IMM group compared to the controls (Two-way ANOVA, p < 0.05 and two-way ANOVA and Mann-Whitney test, p < 0.05, respectively). C5a concentrations are dependent on plasma exudation because complement in milk is not abundant^28^. Accordingly, there were two peaks of C5a concentration at 12 and 24 hpi, which coincided with the first two BSA peaks. The peak at 12hpi was significantly lower in the IMM group and IM group compared to the control group (Two-way ANOVA, p < 0.05).

CXCL3 concentration increased sharply at the onset of the local inflammatory response, and concentrations remained high until 104 hpi. CXCL8 concentrations increased also sharply after 8 hpi, but abated by 40 hpi. There was a difference across immunization groups for CXCL8 concentrations at 12 hpi (Two-way ANOVA and Mann-Whitney test, p < 0.05) for the two immunized groups and 16 hpi for the IMM group only compared to the control and IM groups.

Lactoferrin is another defense protein known to be secreted mainly by MEC during mastitis^29^, and increases in *E. coli* mastitis are known to be delayed and possibly related to the magnitude of the local response^30^. As expected, lactoferrin concentrations increased progressively and reached a plateau by 40 to 48 hpi. There was no difference between the groups in terms of kinetics and concentrations of lactoferrin in milk.

Concentrations of the three major pro-inflammatory cytokines TNF-α, IL-1β and IL-6 increased sharply at the onset of inflammation to reach a peak at 12 hpi. TNF-α disappeared rapidly, but IL-1β and IL-6 remained detectable in milk until 56 hpi. The significant differences across the immunization groups was with TNF-α at 12 hpi with lower concentrations of the IM and IMM groups (Two-way ANOVA and Mann-Whitney test, p < 0.05) and at 16 hpi (Two-way ANOVA, p < 0.05) compared to the control group, and with IL-6 at 12 hpi for the immunized groups and at 24 hpi for the IMM group only compared to the controls (Two-way ANOVA, p < 0.05). At 12 hpi, IL-1β concentration was lower in the IM group and at 24 hpi higher in the IMM group compared to the other groups.

Concentrations of the anti-inflammatory and immunosuppressive cytokine IL-10 increased as soon as 12 hpi and peaked at 24 hpi. Although there was a trend towards an attenuated and delayed IL-10 response in the immunized groups, difference was only significant for the IMM group at 12 hpi (Two-way ANOVA and Mann Whitney test, p < 0.05) and at 16 hpi (Two-way ANOVA, p < 0.01). The main difference in cytokine response was with IFN-γ: concentrations did not rise in control cows, whereas two concentration peaks occurred at 24 and 48 hpi in the milk of immunized cows (Two-way ANOVA and Mann-Whitney test, p < 0.05). Sizeable concentrations of IL-17A were found in the milk of all cows at the onset of inflammation, and concentrations remained elevated after the acute phase with the highest concentration peak at 48 hpi. There was no difference between the groups. Concentrations of IL-22 began to increase also at the onset of the inflammatory response, rose sharply up to 24 hpi, and tended to plateau up to 72 hpi before decreasing progressively. The kinetics of IL-22 differed markedly from the IL-17A concentrations, in particular in terms of concentration persistence. There was no difference between the groups.

### *E. coli* P4-specific antibody and cellular responses upon immunization

**Figure 9:**
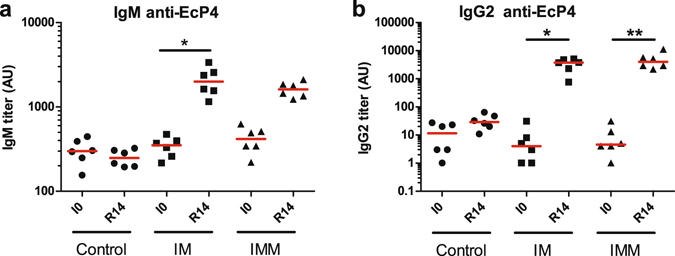
Humoral response to immunisation. Antibody titers were evaluated by ELISA, using HKEc P4 strain as antigen. Results obtained before immunisation (I0) and 2 weeks after the booster immunization (R14) are presented for the adjuvant only (Control), intramuscular (IM) and intramuscular and intramammary (IMM) groups. (**a**) antibody titers in the IgM isotype; (**b**) antibody titers in the IgG_2_ sub-isotype. *p < 0.05, **p < 0.01, Friedman test and post hoc Dunn’s multiple comparison test.

**Figure 10:**
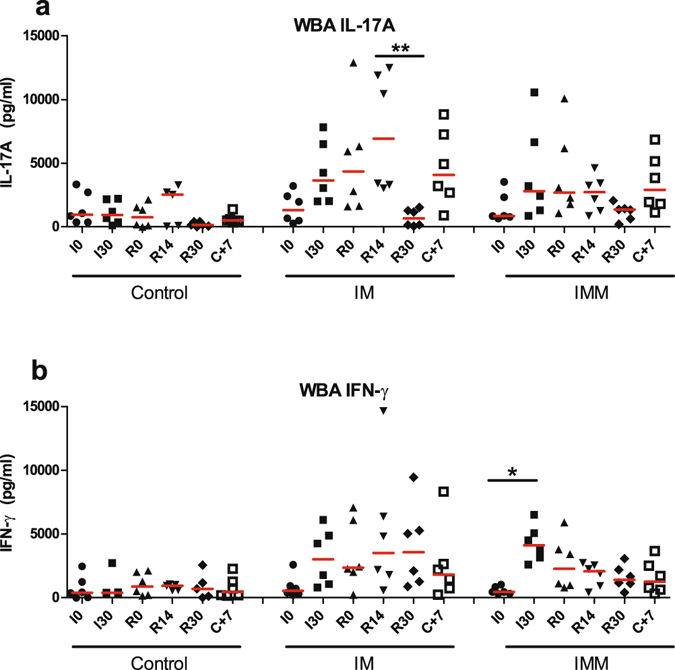
Cellular immune response to immunization. The cell-mediated response was assessed with the whole blood antigenic assay (WBA), using HKEc P4 as antigen. Results obtained before immunization (I0), 1 month later (I30), 2 month after the primary immunization at time of booster injection (R0), 2 weeks after the booster immunization (R14), and 7 days after the infectious challenge (C+7) are presented for the adjuvant only (Control), intramuscular (IM) and intramuscular and intramammary (IMM) groups. (**a**) IL-17A production in the WBA; (**b**) IFN-γ production in the WBA. *p < 0.05 **p < 0.01, Friedman test and post hoc Dunn’s multiple comparison test.

The antibody response elicited by immunization against HKEc P4 was assessed by ELISA in the two isotypes that are opsonic for neutrophils in the bovine species, IgG_2_ and IgM. There was a significant increase in antibody titers in the two isotypes between the first immunization and 14 days after the booster injection in both immunization groups (Fig. 9a and b). We monitored the production of IL-17A and IFN-γ in the WBA because a previous study has shown that there is a good correlation between the production of these cytokines two weeks after an antigenic booster and the neutrophilic response of the mammary gland to local antigen stimulation^9^. The stimulation of whole blood with HKEc P4 induced the production of IL-17A and IFN-γ only in the immunized groups following immunization (Fig. 10). The response was similar in the IM and IMM groups up to the R0 point, as expected (intramuscular injection of killed bacteria in both groups). Following the booster, there was some increase of IL-17A and IFN-γ WBA with the IM group, but not with the IMM group (R14). Of note, the production of IL-17A fell to near baseline levels two weeks later (R30) in both groups. This assay suggests that immunization induced CD4+ circulating T lymphocytes producing IL-17A and/or IFN-γ. Interestingly, the infection induced a recall response in the IL-17A WBA (but not in the IFN-γ WBA) in both groups, 7 days only after the challenge.

### Opsonisation and efficiency of phagocytic killing by neutrophils

**Figure 11:**
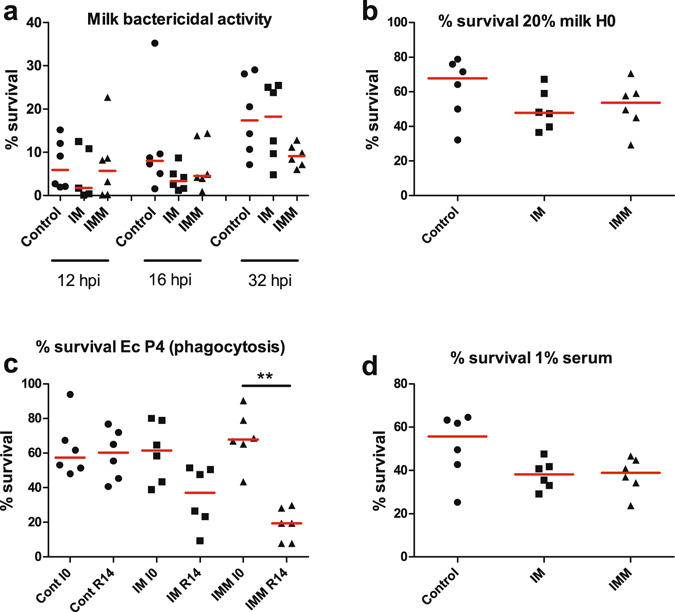
Assessment of the bactericidal activity of whole milk and of the opsonic activity of milk and serum. (**a**) Survival of bacteria (EcP4) after 1 h of incubation under agitation in whole milk drawn from infected glands at 12, 16 or 32 hpi. (**b**) Opsonic activity of milk assessed through the survival of bacteria incubated with blood granulocytes for 90 min under agitation in the presence of 20% milk drawn from glands immediately before challenge (H0). (**c**) Opsonic activity of serum before immunisation (I0) and 2 weeks after the booster immunization (R14), assessed through the survival of bacteria (EcP4) incubated with blood granulocytes for 90 min under agitation in the presence of 0.25% serum. (**d**) Opsonic activity of immune serum (R14) used at 1% in the phagocytic mixture. *p < 0.05, Friedman test and post hoc Dunn’s multiple comparison test.

The *ex vivo* efficiency of the phagocytic killing activity in milk was assessed by comparing the survival of bacteria in whole milk under agitation (phagocytosis) with the survival in milk incubated at rest to take into account possible effects of soluble antibacterial milk components. There was no reduction in cfu numbers in the milk incubated at rest. The efficiency of phagocytosis-dependent bactericidal activity of milk was negligible at 8 hpi (result not shown). A marked reduction in bacterial survival was demonstrated at 12 and 16 hpi (Fig. 11a). The bactericidal activity decreased at 32 hpi in milk of the control and IM cows, but did not abate in milk of IMM cows (Fig. 11a). Nevertheless, the differences between groups were not significant (Kruskal-Wallis test, p = 0.165).

This observation was in keeping with the opsonizing activity of milk and serum. At time of challenge, the opsonizing activity of milk, as assessed by the survival of bacteria incubated with blood neutrophils and 20% milk, did not vary significantly (p = 0.23) between groups despite a slight trend in favor of the immunized cows (Fig. 11b). The exudation of plasma provides opsonic antibodies and thus differences in opsonic titers in serum could result in different opsonic activities in inflammatory milk. This likely happened early in this experiment, as suggested by the steep increase in BSA concentration in milk as soon as 12 hpi (Fig. 8). The sera of the challenged cows proved to be highly opsonic for *E. coli* P4, and it was necessary to lower the serum concentration in the phagocytosis assay to discriminate the sera. At the final concentration of 0.25%, sera proved to be more efficient at opsonizing *E. coli* P4 after immunization, and the difference was significant (p < 0.01) with the sera of cows of the IMM group (Fig. 11c). This most likely reflects the increase in antibody titers in the opsonic isotypes IgG_2_ and IgM. Nevertheless, the difference in opsonic activity was not demonstrated when the concentration of serum was increased to 1% (Fig. 11d), suggesting that during infection the slightly different opsonic activities were not of biological significance in the milk of infected quarters, whatever the immunization group.

## Discussion

This experiment was designed to investigate the modifications induced by immunization through two different routes on the course of *E. coli* mastitis, with a view to increasing our understanding of the mechanisms that contribute to immune protection of the mammary gland. Overall the course of infection in control cows was similar to that previously reported in experiments carried out with the P4 strain^12, 13, 31^. Challenged cows experienced a clinical mastitis with local and systemic signs, characterized by an initial and transient acute phase followed by a sustained local inflammatory response. Bacteria proliferated in milk until leukocytes, mainly neutrophils, were mobilized en masse to reach the lumen compartment of the mammary gland. As usual in primiparous healthy cows, the infection is cleared in a few days, and the local inflammation abates progressively^32^. Overall, the severity of infections induced by the challenge was representative of most clinical coliform mastitis, eliciting local acute phase protein SAA concentrations comparable to those published elsewhere^33^.

As anticipated, the course of infection and the inflammatory response were impacted by immunization. The three groups of cows differed in their response to infection in several respects: systemic and local clinical signs, kinetics of bacterial clearance, and milk cytokine profile. The protocol involving local immunization was the most effective in reducing the severity of mastitis. A major impact of immunization was the early reduction in *E. coli* concentration in milk and earlier elimination of bacteria (Fig. 4). Interestingly, the improved antibacterial efficiency of the immune response was not associated with an increased inflammatory response. Overall, reduced infection was concomitant with reduced systemic inflammatory response. In clinical terms, IMM immunization reduced the systemic response compared to the other two groups, with the exception of the febrile response, which was prolonged in the immunized groups with a second episode at 48 hpi (Fig. 2). The prolonged clinical response was also apparent locally, especially in the IM group, as shown by the clinical scores and the ultrasonographic measurements (Fig. 3), but overall the local clinical signs did not differ across the groups (Fig. 3). The reduction in systemic signs was paralleled by a reduction in neutropenia and lymphopenia.

The favorable change of the course of infection was obtained with a modification of the milk cytokine profile. The major difference was the absence of IFN-γ in the milk of control cows, whereas immunized cows displayed a marked increase in IFN-γ concentrations, in particular in the IMM group at the onset of inflammation (Fig. 8). This increase in local IFN-γ production is likely to have contributed to the improved bacterial clearance, as cows treated intramammarily with IFN-γ have enhanced resistance to mastitis^34, 35^. The other main difference was the reduction in the transient burst of TNF-α concentration in the milk of immunized cows. It is likely that this reduction contributed to the general trend of reduced inflammation indicators in immunized cows, such as blood haptoglobin concentrations or serum albumin and SAA concentrations in milk, because the production of TNF-α during acute *E. coli* mastitis has been shown to be associated with the severity of clinical symptoms^36^. It is also possible that the increase in IFN-γ and the reduction in TNF-α concentrations were related, as the intramammary infusion of recombinant bovine IFN-γ prior to experimental infection with *E. coli* prevented the increase in TNF-α concentrations in milk and serum^36^. The reduced TNF-α concentrations are not likely to result from a reduced infection stimulus, because the bacterial load was similar in all groups during the first 24 hpi (Fig. 4).

A novel observation was the early and sustained production of IL-17A during *E. coli* mastitis (Fig. 8). Taking into account the dilution by several liters of milk, this amounted to a sizeable production, even though the highest concentrations reached a peak at 48 hpi when milk production was at its lowest. Unexpectedly, there was no difference in IL-17A concentrations between groups. The immunization protocol had been devised with the intention of eliciting a Th1/Th17 type immune response, based on previous work showing that this type of response can be induced with the adjuvant used and monitored with a whole blood antigen assay^9^. The whole blood assay showed that there was indeed some induction of circulating *E. coli*-specific IL-17A- and IFN-γ-producing cells two weeks after the booster immunization, in particular in the IM group (Fig. 10). It is possible that the immunization schedule we used was not optimal, so that the induction of Th17 cells was of low magnitude, and the homing of lymphocytes to the mammary gland may not have been efficient enough. More comprehensive data on the immune response to immunization are being analyzed and will be presented in a following article. Notwithstanding, it seems likely that the IL-17A measured in milk was produced by innate immune cells, such as γδ T cells or innate lymphoid cells like natural killer (NK) cells or even neutrophils^37–39^. Another new observation was the induction of IL-22 production in milk by the infection. Concentration increases were progressive if compared to those of TNF-α or IL-17A, but concentrations remained high beyond 64 hpi, a time when all the other measured cytokines had returned near to baseline (Fig. 8). This protracted secretion may be related to the wound healing activity attributed to IL-22, if we suppose that IL-22 contributed to the mammary gland recovery following *E. coli* mastitis, besides its recognized contribution to antimicrobial defense of epithelial barriers^40^. As there was no difference induced by immunization, we can surmise that IL-22 was mainly produced by innate immune cells^41^. The difference of secretion kinetics from that of IL-17A suggests that these two cytokines were produced by different cells, or were at least under different regulation pathways. To determine the cellular sources of IL-17A and IL-22 in the infected mammary gland should be a rewarding research task in the future.

To what defense mechanism could we attribute the improved efficiency of the response of the mammary gland to infection achieved in our study? It is generally accepted that opsonophagocytic killing by neutrophils plays a major role in *E. coli* mastitis^42^. That is why we measured a number of indicators of the efficiency of this major defense mechanism. An unexpected result was the absence of reinforced recruitment of leucocytes into milk at the onset of the inflammatory response. On the basis of the Th1/Th17 profile previously achieved with this immunization protocol^9^, and a previous observation with an intramammary immunization with *E. coli* ^26^, an initial increase in cell recruitment in milk was anticipated due to the synergy between innate and adaptive responses for the influx of neutrophils in the mammary gland^43^. On the contrary, control cows tended to recruit more leukocytes at the initial stage of inflammation (Fig. 5a). This lack of reinforced mobilization of leukocytes is in keeping with the observed trend towards a reduction in the neutrophil chemoattractant concentrations (C5a, CXCL3, and CXCL8) in milk of immunized cows at the onset of inflammation (Fig. 8). Among leucocytes, neutrophils were massively recruited into milk, but at the onset of the inflammatory response (12 hpi) neutrophil recruitment was more intense in the milk of control cows (Fig. 5c). This is in keeping with higher concentrations of CXCL8 in the milk of control cows at this time, and also of CXCL3 and C5a (Fig. 8), which are potent chemoattractants for bovine neutrophils^44, 45^. C5a concentration peaks at 12 and 24 hpi coincided with the first two BSA concentration peaks, which can be explained by the necessity of plasma exudation to reach high complement concentrations in milk. The absence of a third C5a concentration peak concomitant with the third peak of BSA concentration most likely resulted from a weak complement activation by too few remaining bacteria in milk. Apart from the first concentration peak at the onset of the inflammatory response, concentration peaks of BSA coincided with concentration nadirs of CXCL3 (Fig. 8). Intriguingly, BSA concentration peaks occurred 8 h after a complete milking, whereas CXCL3 concentrations occurred 16 h after a complete milking. It can be hypothesized that milking favored plasma exudation, thus BSA concentration in milk, whereas CXCL3, which is mainly produced by mammary epithelial cells^19^, accumulated in milk between milking times.

Increased opsonic activity is considered to favor phagocytosis and killing by neutrophils^46^, and immunization increased the opsonic titers to some extent (Fig. 11c). There was also more IFN-γ in the milk of immunized cows, and IFN-γ has been shown to increase the bactericidal activity of bovine PMN^47, 48^. These conditions did not result in improved killing efficiency in the *ex vivo* assay (Fig. 11a). Thus, there is no indication for the better control of infection to be related to improved opsonophagocytic killing.

The absence of effect of immunization on the opsonophagocytic efficiency of neutrophils occurred although the homologous strain has been used for both immunization and challenge. It is known that most opsonic antibodies induced by *E. coli* are directed to the polysaccharide portion of LPS, and consequently are mainly serotype-specific. It is generally accepted that O-specific antibodies offer superior protection against homologous smooth Gram-negative bacterial strains^49–52^. In our study, homologous challenge is not likely to have had a determinant effect on the outcome of infection, as far as antibodies and opsonins are concerned. Our results are in keeping with the idea that opsonic antibodies did not play a major role in the favorable effect of immunization on the outcome of infection. Nevertheless, the specificity of the antibody response warrants further study. This matter will be further investigated in a following in-depth study of the specificity of induced antibodies and their opsonic activity towards *E*. *coli* mastitis strains of different serotypes.

Amongst the mechanisms that may lead to protection, the role of tissue-resident memory cells that may have been recruited or educated through local immunization remains to be determined. Systemic immunization may have produced a pool of memory circulating *E. coli*-specific CD4^+^ T cells, that was further associated with establishment of tissue-resident memory cells in the mammary microenvironment after the local recall injection. This pattern of response has been primarily devised for CD8 T cells^53^, but is now also recognized for the protection mediated by CD4 T cells^54^; this may explain the biphasic response that was observed in immunized groups, as previously described for the genital tract in a mouse vaccine model^55^. Unexpectedly, there was no recall response to the booster immunization in the IMM group as assessed with the WBA (Fig. 10). The WBA can only detect circulating antigen-specific lymphocytes. The prime-boost protocol used in our study was designed to induce a first wave of lymphocytes colonizing the mammary tissue (sometime after the peak of WBA positivity), then to boost the local response locally. We do not know whether the local booster can induce circulating antigen-specific lymphocytes, and consequently we cannot state over the efficiency of the booster on the basis of the WBA. The answer to this issue resides in the mammary tissue, and the analysis of tissue samples would be necessary to substantiate a local booster effect. Interestingly, the induced infection was followed by an increase in the production of IL-17A in the WBA for both immunized groups (Fig. 10). The absence of such a response in the control group is in agreement with the induction of an adaptive response to immunization.

A limitation of the study is the lack of further characterization of the lymphoid cells recruited in milk. This was precluded by the intense recruitment and activation of neutrophils which self-aggregated and co-aggregated lymphocytes. Although the identification of the lymphocyte populations recruited in milk during mastitis would have yielded interesting results, these cells are not necessarily representative of those which triggered the response from within the tissue. Further experiment involving tissue sampling of sacrificed cows shortly after infection is likely to shed some light on the cells which in the mammary tissue have a major role in the defense to infection. Another opportunity offered by the analysis of tissue specimens would be to account for the contribution of antimicrobial peptides to the vaccine-induced immune response to *E. coli* infection. Antimicrobial peptides, such as Lingual Antimicrobial Peptide, are induced during mastitis^56^, but their contribution to protection during *E. coli* mastitis remains to be established. In our study the antimicrobial effect of whole fresh milk was shown to be negligible (control of the opsonophagocytosis assay), suggesting that antimicrobial peptides did not play an important role in the control of *E. coli* growth in milk.

Intramammary immunization has previously been investigated as a procedure to reduce the clinical signs of coliform mastitis, in particular by using the *E. coli* J5 vaccine, with variable success. In one study, the intramammary route was efficient in inducing antibodies to *E. coli* J5 and the *E. coli* strain used for intramammary challenge, but not for reducing the severity of clinical signs and milk losses^57^. In another study, intramammary immunization with *E. coli* J5 was related to some protection against challenge with a mastitis *E. coli* isolate, characterized by a reduction of clinical symptoms and a lower probability of infection^58^. This result is surprising because it was obtained with intramammary immunization with only 100 cfu of UV-killed bacteria, which is a very minute amount of antigen. Also, the challenge was carried out in dry glands, about 10 days before the expected calving date, with a strain isolated from a mild persistent intramammary infection. As the bacterial numbers in mammary secretion remained very low (<10 cfu) and leucocyte counts following challenge were not reported, it is difficult to determine if an infection resulted from the challenge. The prevention of *E. coli* intramammary infection without inflammatory response would be an unprecedented success, but needs to be confirmed in lactation with representative mastitis-causing *E. coli* strains.

Besides adaptive immunity evidenced by the increase in milk concentration of IFN-γ and the increase in the IL-17A WBA after infection, the intramammary booster immunization may have induced an innate immune response. There are a few data relating to such a possibility in the mammary gland, such as the alleviation of the inflammatory response 10 days after the intraluminal infusion of LPS^59^. In this experiment, local injection of 1 µg LPS improved the response to *E. coli* experimental mastitis. There is also evidence that some mammary gland tolerance to *E. coli* infection lasts at least 2 weeks^33^. In our case, the duration of innate “memory” or tolerance should have been more than 80 days (timespan between the booster and the challenge). This is not impossible, as far as epigenetic changes are involved, but this has never been substantiated in the context of mastitis.

In conclusion, local immunization modified favorably the course of infection, by limiting the inflammation while accelerating bacterial clearance. This was obtained with a modification of the cytokine profile but not by an increase in the initial efficiency of opsonophagocytic activity in milk. There was a sizeable and sustained production of IL-17A and IL-22 in milk, independent of the immunization status, and thus likely to be of innate origin. The observed reduction in mastitis symptoms and improvement of resistance to infection consecutive to immunization is likely to be related to the induction of IFN-γ and reduction in TNF-α production. Overall, these results suggest that the humoral response to *E. coli* mastitis vaccine is not of prime importance and that cell-mediated immunity is the key to understanding vaccine-induced protection of the mammary gland. They also suggest that the immunization schedule used in our study may still be improved with a view to inducing a Th17-type immune response. This prompts further studies to determine how to induce and orient such a response in the cow.

## Electronic supplementary material

Supplementary Information
