# Supplementary material for: Local immunization impacts the response of dairy cows to Escherichia coli mastitis
Source: Sci Rep. 2017 Jun 13;7:3441. doi: 10.1038/s41598-017-03724-7 (PMC5469773; doi:10.1038/s41598-017-03724-7)
Supplement: Supplementary file 1 — Supplementary Information [file 41598_2017_3724_MOESM1_ESM.pdf]

Local immunization impacts the response of dairy cows to *Escherichia coli* mastitis

Vincent Herry, Christophe Gitton, Guillaume Tabouret, Maryline Répérant, Laurine Forge, Christian Tasca, Florence B. Gilbert, Edouard Guitton, Céline Barc, Christophe Staub, David G.E. Smith, Pierre Germon, Gilles Foucras, and Pascal Rainard

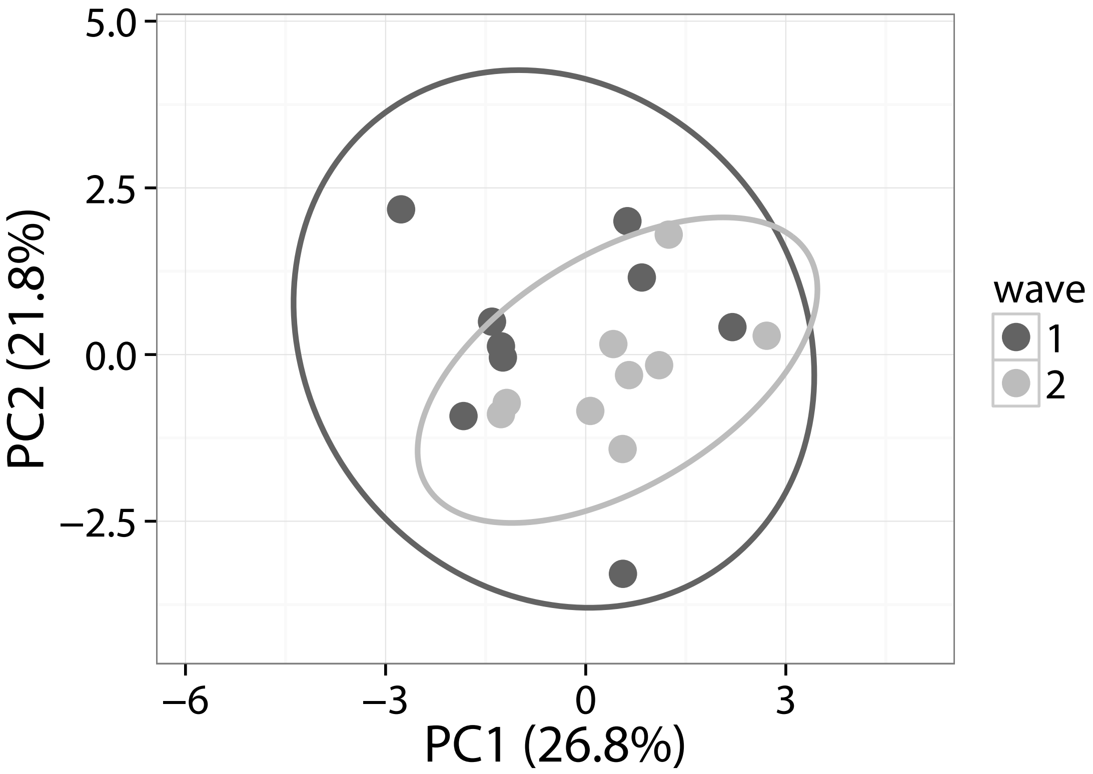

Supplementary Figure S1. 2D Principal Component Analysis of the results of the experimental mastitis challenge on 18 cows according to the wave. Eleven parameters (systemic clinical score, milk production, SCS, bacteriolo-gy, cytokine production) that were significantly different amongst groups were used to construct a matrix for PCA. Unit variance scaling is applied to rows; SVD with imputation is used to calculate principal components. X and Y axis show principal component 1 (PC1) and principal component 2 (PC2) that explain 26.8% and 21.8% of the total variance (parentheses), respec-tively. Each point represents a different cow. Prediction ellipses are such that with probability 0.95, a new observation from the same group will fall inside the ellipse.

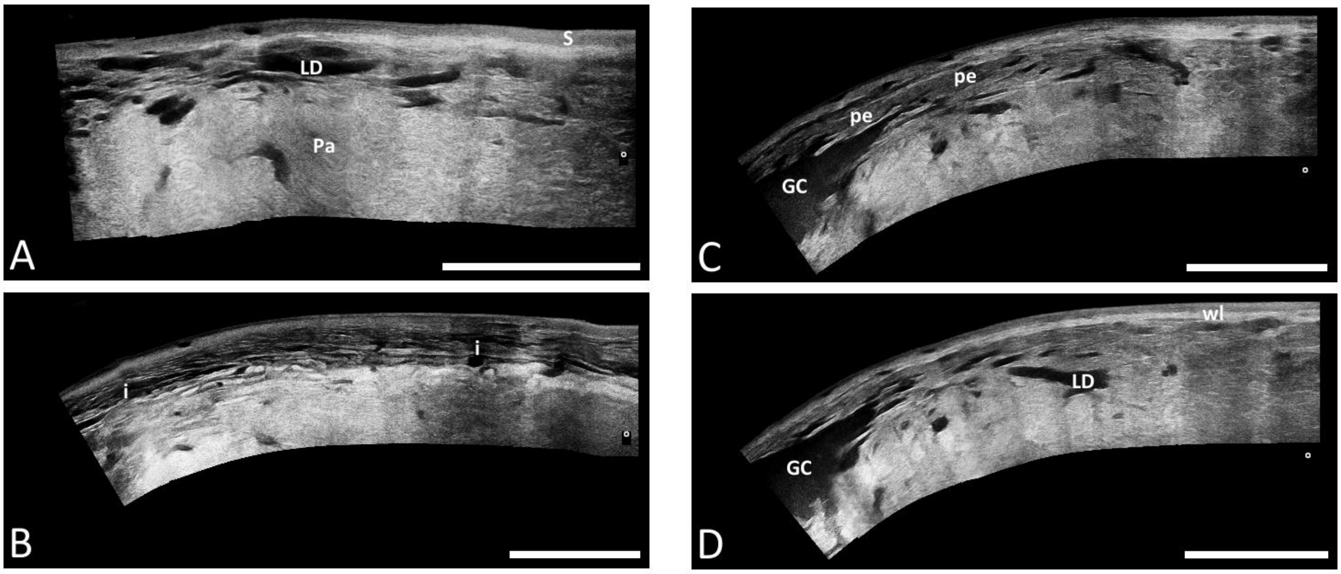

Supplementary Figure S2. Ultrasound photographs of the udder gland parenchyma before *E.Coli* intramammary infusion (A; T0) and post infusion while subcutaneous inflammation is developing (B; T2: 16h) and regressing (C; T3: 32h and D; T6: 80h). The top of the udder is on the right side of the photographs. At the bottom of the udder, gland cistern (GC) connected to the teat is on the left side of the photographs. (A) Udder parenchyma is bright (echogenic) and homogeneous (S: skin; LD: lactiferous duct; Pa: parenchyma). (B) The dark area below the skin represents the subcutaneous inflammation (i: inflammation). (C) Inflammation is regressing but permeabilisation of the parenchyma is still noticeable (pe: permeabilisation) as shown by the dark (low echogenic) subcutaneous tissue. (D) Udder parenchyma is back to normal but a subcutaneous white line (wl) is still betraying the former inflammation. In all photographs the bar represents 5cm.
